# Shifting perceptions of female genital cutting in a Swedish migration context

**DOI:** 10.1371/journal.pone.0225629

**Published:** 2019-12-04

**Authors:** Anna Wahlberg, Sara Johnsdotter, Katarina Ekholm Selling, Birgitta Essén

**Affiliations:** 1 Department of Women’s and Children’s Health, International Maternal and Child Health, Uppsala University, Uppsala, Sweden; 2 Faculty of Health and Society, Malmö University, Malmö, Sweden; Social & Preventive Medicine Institute, SWITZERLAND

## Abstract

**Background:**

The aim of this paper was to investigate correlations between Somali Swedish own attitudes towards female genital cutting (FGC) and their perceptions about other Swedish Somalis attitudes.

**Methods:**

In 2015, a cross-sectional study was conducted in four Swedish municipalities with 648 Somali men and women. To assess the level of agreement between the participants’ approval of FGC and their *perceptions* about approval among other Swedish Somalis, Bangdiwala’s B-statistic and Welch’s *t*-test were used.

**Results:**

We found a substantial agreement between an individual’s own approval of FGC and their perceived approval of FGC among most other Swedish Somali men (B-statistic = 0.85) and women (B-statistic = 0.76). However, we also found a tendency for participants to report that other Swedish Somalis–and especially other Swedish Somali women–approved of FGC, while they themselves did not. Perceived percentage of Somali girls being circumcised in Sweden was significantly higher among Swedish Somalis who said they wanted tissue to be removed on their own daughter (mean 23%, 95% CI: 18.3–27.9) compared to those who said they opposed removal of tissue on their own daughter (mean 8%, 95% CI: 6.4–9.1). The majority of Swedish Somali men (92%) stated a preference to marry someone without FGC or with pricking, which was also the view of most of the Swedish Somali women (90%).

**Conclusions:**

Swedish Somalis motivation to continue or discontinue with the practice of FGC may be influenced by perceptions of what other Swedish Somalis prefer. How FGC is being portrayed, in for example media reports, could therefore have an impact on attitudes towards FGC.

## Introduction

With globalisation, people from female genital cutting (FGC) practising countries resettle in countries that traditionally do not practise FGC on girls. Consequently, governments and international organisations are increasingly concerned about the risk of FGC in immigrant populations in traditionally non-practising countries, such as Sweden [1,2].

Approximately 38,000 circumcised women live in Sweden, of which 21,000 (55%) are from Somalia [2]. For a Swedish resident, it is illegal to perform or make arrangements for the performance of any form of FGC, both in Sweden and abroad [3]. Research suggests that the support for FGC among Somali men and women in Sweden is low [4–6], while in Somalia, the prevalence of FGC is close to 100% [7,8]. The most common type of FGC in Somalia is infibulation; however, less extensive forms of FGC appear to have become more accepted [9].

To improve the success of programmes that target the practices of FGC in countries where they are prevalent, theories of behaviour change in general, and social norms theory in particular, are increasingly incorporated within these programmes. In a model first developed by Gerry Mackie, it was proposed that the continuation of FGC is interdependent and upheld by social norms related to marriageability, and that compliance with such norms is important to ensure the acceptance of a girl and her family in the community [10]. In several of the UNICEF reports, social norms theory is explored from a theoretical and behaviour change perspective based on programme experiences in FGC-practising countries [11–13]. In addition, scientific literature on social norms, influence and FGC has provided insight about the fluidity of social norms [14] and the way in which structural, interpersonal, and individual aspects interact and shape decision making regarding FGC [15–19].

It has been suggested that social norms theory can also be applicable in a migration context [4,11]. Knowledge of the influence of social norms in a migration context is important as it may help us to understand the dynamics of attitude and behaviour change among immigrants, as well as to inform the design of interventions to be implemented in a migration context.

The relation between attitudes, norms and conventions is complex. First, social norms are constellations of normative attitudes. Second, there is a connection between social norms and how individuals behave, as well as between social norms and *beliefs* about how other people behave. Thus, where there is a social norm, individuals will generally want to act in accordance with the norm, presuming enough others also do. Third, individuals will, in general, behave in accordance with conventions, as well as *believed* conventions [20]. In relation to the practices of FGC, an individual’s behaviour has been shown to be connected with attitudes and norms towards FGC [21,22].

This study aimed to investigate correlations between Somali Swedish own attitudes towards FGC and their perceptions about other Swedish Somalis attitudes in order to grasp possible changes of social norms and the convention of FGC in a migration context.

## Methods

### Hypotheses

In this study, we assessed the following hypotheses:

1. The perception that other community members approve of FGC correlates with the individuals’ propensity to support FGC.
2. The perception that other girls in the community will be circumcised correlates with the individuals’ propensity to support FGC on a (hypothetical) daughter.
3. The convention to circumcise in order to enhance marriageability is not found in a Swedish migration context.

### Data collection and participants

This was a cross-sectional study conducted in four Swedish municipalities in 2015. A 49-item questionnaire–that had been pilot-tested, validated, and translated and back-translated from English to Somali–was used to collect data (S1 and S2 Files). Six Somali research assistants, three men and three women, informed the study’s design, questionnaire development and interpretation of the findings. Further, they recruited participants and collected data through face-to-face interviews in Somali using the questionnaire. The research assistants interviewed both men and women, regardless of their own gender. Using face-to-face interviews ensured that illiterate Somalis could also participate in the study. The interviews were conducted in a private setting, and the participants were informed that the information they provided would be treated with confidentiality. As a method of quality control, the first author (AW), together with the research assistants, looked through and discussed the answers in the majority of the questionnaires. If inconsistencies or missing data were found, the participant was contacted for clarification. In addition, we also conducted two workshops with approximately 30 and 60 Somali immigrants, respectively, in two study areas in order to validate the findings.

Eligible to participate were Somali-born men and women living in Sweden, aged 18 years or older. The majority of the participants were recruited through purposeful sampling primarily at Somali organisations, but also in public places such as cafés, at Swedish for Immigrants courses, and in mosques. Some participants were recruited through snowball sampling. Oral informed consent was obtained and documented on a checklist for all participants, as approved by the ethical review board. The Regional Ethical Review Board of Uppsala, Sweden approved the study (2014/274).

### Measurement of type of FGC

Questions relating to FGC were based on the anatomical extent of FGC, which can roughly be divided into: 1) pricking, no tissue removed; 2) some tissue removed; 3) tissue removed, some stitching; and 4) tissue removed and narrowing of the vaginal orifice through stitching of the cut labia. To not restrict ourselves (and the participants) to these four categories of FGC, attitudes towards FGC were measured on Visual Analogue Scales (VAS) ranging from 0 to 100 millimetres (mm) to capture all forms of FGC. In the questionnaire, the left end of the VAS (0 mm) was marked with ‘Nothing at all’ and the right end (100 mm) with ‘Flesh removed and closed’ [5]. Thus, with increased millimetres on the VAS, the anatomical extent of FGC increased. The data collectors were responsible for ensuring that the participants accurately understood the different anatomical forms of FGC and, by using a schematic picture describing the different anatomical forms of FGC, assisting the participants in expressing their attitudes on the VAS.

After data collection, we dichotomised the VAS measurement into the anatomical impact of the practice. Answers ranging from 0–10 mm on the VAS were defined as ‘no removal of tissue’ and include untouched genitals (0 mm) and pricking of the skin with no removal of tissue (1–10 mm). We decided to adopt this conservative definition of pricking (1–10 mm, rather than, e.g., 1–25 mm) so as not to underestimate the number of participants who support the removal of tissue. Answers ranging from 11–100 mm were defined as ‘removal of tissue’ and included forms of FGC where tissue is removed with/without stitching. We chose this anatomical classification as we found that some participants thought that, for a practice to be considered as FGC, tissue needed to be removed [6]. However, we also present data where attitudes supporting pricking are not grouped together with attitudes supporting no form of FGC.

### Variables

To test hypotheses 1 and 2, we analysed the agreement between the participants’ approval of FGC and their perceptions about approval among most other Somalis living in Sweden. To test hypothesis 3, measurements of whether Swedish Somali men preferred to marry someone with FGC and what women believed men preferred were used (Table 1). Background variables included gender (man, woman), age (18–25, 26–35, 36–45, ≥ 46), years of residency in Sweden (≤ 2, 3–4, 5–9, 10–14, ≥ 15), marital status (single, married/partner, divorced/widowed), education (university/college, secondary school, primary school, Koranic school, no education), and Somali origin (rural, urban).

**Table 1:** Variables used to test the hypotheses that approval of FGC correlates with perceptions about what others prefer.

Original question (Somali translation in questionnaire)	Variables	Categorisation^a^
**Hypothesis 1: The perception that other community members approve of FGC correlates with the individuals’ propensity to support FGC**		
*What do you think is acceptable to do*?	Approval of FGC	no tissue removed nothing (0 mm) pricking (1–10 mm) tissue removed (11–100 mm)
*What do you think most Somali men in Sweden think is acceptable to do*?	Perceived approval of FGC among Swedish Somali men	no tissue removed nothing (0 mm) pricking (1–10 mm) tissue removed (11–100 mm)
*What do you think most Somali women in Sweden think is acceptable to do*?	Perceived approval of FGC among Swedish Somali women	no tissue removed nothing (0 mm) pricking (1–10 mm) tissue removed (11–100 mm)
**Hypothesis 2: The perception that other girls in the community will be circumcised correlates with the individuals’ propensity to support FGC on a (hypothetical) daughter**		
*We don’t know if you have a daughter*. *But let’s hypothetically say that you do have a daughter*, *what would you then do*?	Preferred form of FGC on hypothetical daughter	no tissue removed nothing (0 mm) pricking (1–10 mm) tissue removed (11–100 mm)
*How many within the Somali community in Sweden do you think circumcise their daughters*?	Perceived percentage of Somali girls being circumcised in Sweden	0–100%
**Hypothesis 3: The convention to circumcise in order to enhance marriageability is not found in a Swedish migration context**		
*Men*: *For your marriage*, *do you prefer a woman who is circumcised or one who is not circumcised*?	Men: marriage preference	no tissue removed not circumcised pricking but no tissue removed tissue removed some tissue removed tissue removed and some stitching tissue removed and closed does not matter
*Women*: *Do you think Somali men prefer to marry a woman who is circumcised or one who is not circumcised*?	Women: perceived marriage preference among Somali men	no tissue removed not circumcised pricking but no tissue removed tissue removed some tissue removed tissue removed and some stitching tissue removed and closed does not matter

^a^VAS measurement in millimetres within the brackets

### Sample size

Two sample size calculations were made based on two continuous variables: the participants’ approval of different forms of FGC; and the participants’ preferred form of FGC on a hypothetical daughter, using the formula n = ((1.96*SD)/precision)^2^. The standard deviation (SD) for these two variables was based on the first 107 collected questionnaires (10.42 and 17.84, respectively), and precision of the 95% confidence interval was chosen at 2.5. To adjust for the design effect, the two estimates were thereafter each multiplied by 2.25 which is the average value of the design effect for the DHS indicators [23]. Of the two calculations, the highest estimate gave a required sample size of 441.

### Statistical methods

Descriptive statistics are presented as frequencies and percentages. To assess the level of agreement between the participants’ approval of FGC (‘no tissue removed, ‘tissue removed’) and the perceived approval among most other Swedish Somali men and women (‘no tissue removed, ‘tissue removed’) we used Bangdiwala’s B-statistic and its corresponding agreement chart (presenting the marginal totals) [24]. This measurement is recommended instead of Cohen’s kappa when the marginal distribution is imbalanced, which was the case with our data. However, as the B-statistic is a less common measure, we also report Cohen’s kappa. Both statistics quantify the agreement after correcting for the agreement that arises from chance alone. Bangdiwala’s B-statistic is defined from the agreement chart and was calculated as the ratio of the sum of areas of squares of perfect agreement to the sum of areas of rectangles of marginal totals. Cohen’s kappa was calculated by: (Observed agreement—expected agreement)/(1—expected agreement) [24,25]. Welch’s *t*-test was used to determine the difference in means in how common, in percentage, the participant thought it was for Somali girls to be circumcised in Sweden (continuous variable), depending on the participants’ own preferred form of FGC on a hypothetical daughter (‘no tissue removed, ‘tissue removed’). A *p*-value of less than 0.05 was considered statistically significant. SPSS version 23 and the ‘vcd’ package [26] in RStudio version 1.0.44 [27] were used for all statistical analyses.

## Results

Table 2 presents characteristics of the 648 Somali men and women living in Malmö (203, 31%), Gothenburg (188, 29%), Stockholm (175, 27%), and Uppsala (82, 13%). The mean age was 38 years, ranging from 18–73 years and the vast majority (>99%) were Muslims. Among the women, the most common self-reported form of FGC was tissue removed and closed (infibulation) (41%), followed by tissue removed and some stitching (25%), some tissue removed (12%), pricking (7%), untouched (2%), and unspecified (13%).

**Table 2:** Descriptive statistics of background factors among Somali immigrants in four municipalities in Sweden (*n* = 648), 2015.

	*n*/N	%
**Gender**		
Man	330/648	50.9
Woman	318/648	49.1
**Age**		
18–25	91/642	14.2
26–35	217/642	33.8
36–45	166/642	25.9
≥ 46	168/642	26.2
**Years of residency in Sweden**		
≤ 2	168/647	26.0
3–4	111/647	17.2
5–9	163/647	25.2
10–14	80/647	12.4
≥ 15	125/647	19.3
**Marital status**		
Single	246/644	38.2
Married/partner	343/644	53.3
Divorced/widowed	55/644	8.5
**Education**		
University/college	62/644	9.6
Secondary school	207/644	32.1
Primary school	257/644	39.9
Koranic school	43/644	6.7
No education	75/644	11.6
**Somali origin**		
Urban	521/642	81.2
Rural	121/642	18.8

### Hypothesis 1. The perception that other community members approve of FGC correlates with the individuals’ propensity to support FGC

We found a moderate to substantial agreement between the participants’ own approval of FGC and their perceived approval of FGC among most other Swedish Somali men (B-statistic = 0.85, kappa = 0.54) and women (B-statistic = 0.78, kappa = 0.40) [28,29] (Table 3). Further, the expected agreement to be present by chance alone between participants’ own ‘approval of FGC’ and ‘perceived approval among Swedish Somali men’ was 0.74, and the observed agreement was 0.88, indicating a greater agreement than expected by chance. Similarly, the expected agreement between own ‘approval of FGC’ and ‘perceived approval among Swedish Somali women’ was 0.70, and the observed agreement was 0.82.

**Table 3:** Cross-tabulations comparing the agreement between Swedish Somalis’ own approval of FGC and their assumptions about approval of FGC among most other Swedish Somali men and women.

	Approval of FGC	
	No tissue removed (%)	Tissue removed (%)
**Perceived approval among Swedish Somali men**^a^		
No tissue removed	507/572 (88.6)	14/74 (18.9)
Tissue removed	65/572 (11.4)	60/74 (81.1)
**Perceived approval among Swedish Somali women**^b^		
No tissue removed	471/571 (82.5)	17/74 (23.0)
Tissue removed	100/571 (17.5)	57/74 (77.0)

^a^ Bangdiwala’s B-statistic 0.85, Cohen’s kappa 0.54

^b^ Bangdiwala’s B-statistic 0.78, Cohen’s kappa 0.40

**Fig 1:**
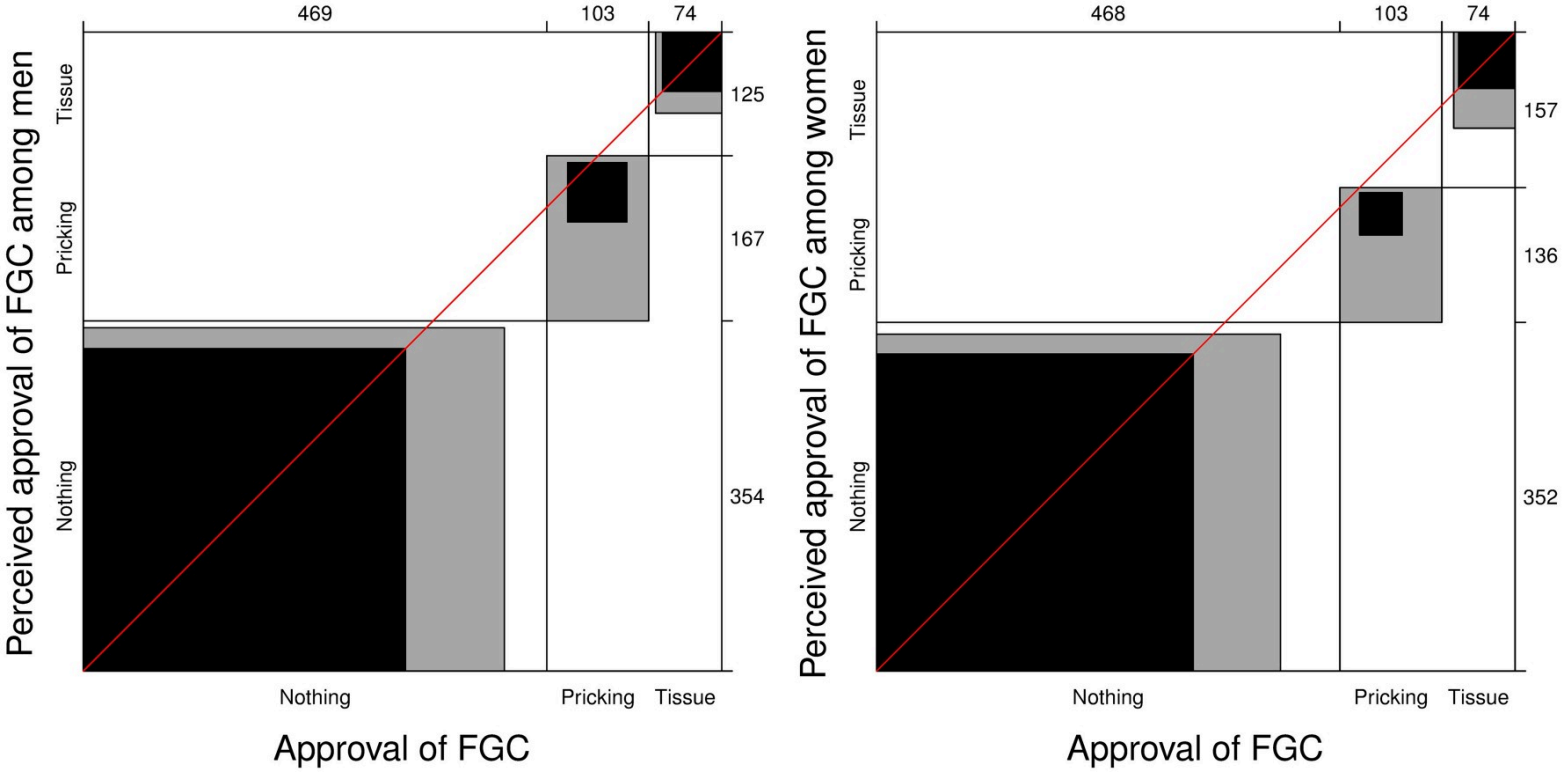
Agreement charts illustrating the agreement between Swedish Somalis approval of ‘no circumcision’, ‘pricking’ or ‘removal of tissue’ (x-axis) as compared with their assumptions about approval among most other Swedish Somali men (left graph) and women (right graph) (y-axis). Within the rectangles, the black area represents complete agreement, the grey area partial agreement, and the white area no agreement.

Fig 1 illustrates the relationship between Swedish Somalis’ own approval of FGC compared with their *assumptions* about what forms of FGC most other Somali men and women in Sweden approve of. The figure is based on the number of participants who reported that they did not approve of any form of FGC (469/647, 73%), those who expressed an approval of pricking (103/647, 16%), and those who said they approved of removal of tissue (75/647, 12%), compared with the participants’ perceived approval of FGC among Swedish Somali men and women. The participants’ perception of Swedish Somali men was that 55% (354/646) would not approve of any form of FGC, 26% (167/646) would approve of pricking, and 19% (125/646) would approve of the removal of tissue. The participants’ perception of Swedish Somali women was that 55% (352/645) would not approve of any form of FGC, 21% (136/645) would approve of pricking, and 24% (157/645) would approve of the removal of tissue. Within the rectangles in the figure, the black area refers to complete agreement, the grey area to partial agreement, and the white area to no agreement. The black area is substantially larger than the grey and white areas, which indicates a good agreement between Swedish Somalis’ own approval of FGC and how they perceive other Swedish Somalis’ approval. In the case of perfect agreement, the intersections of the rectangles would not deviate from the 45° diagonal line. However, for both charts in the figure, the intersections are below the diagonal line. This means that Swedish Somalis to a larger extent said that they perceived that other Swedish Somalis–and especially other Swedish Somali women–approved of the removal of tissue, while they themselves did not approve of any form of FGC.

### Hypothesis 2. The perception that other girls in the community will be circumcised correlates with the individuals’ propensity to support FGC on a (hypothetical) daughter

**Fig 2:**
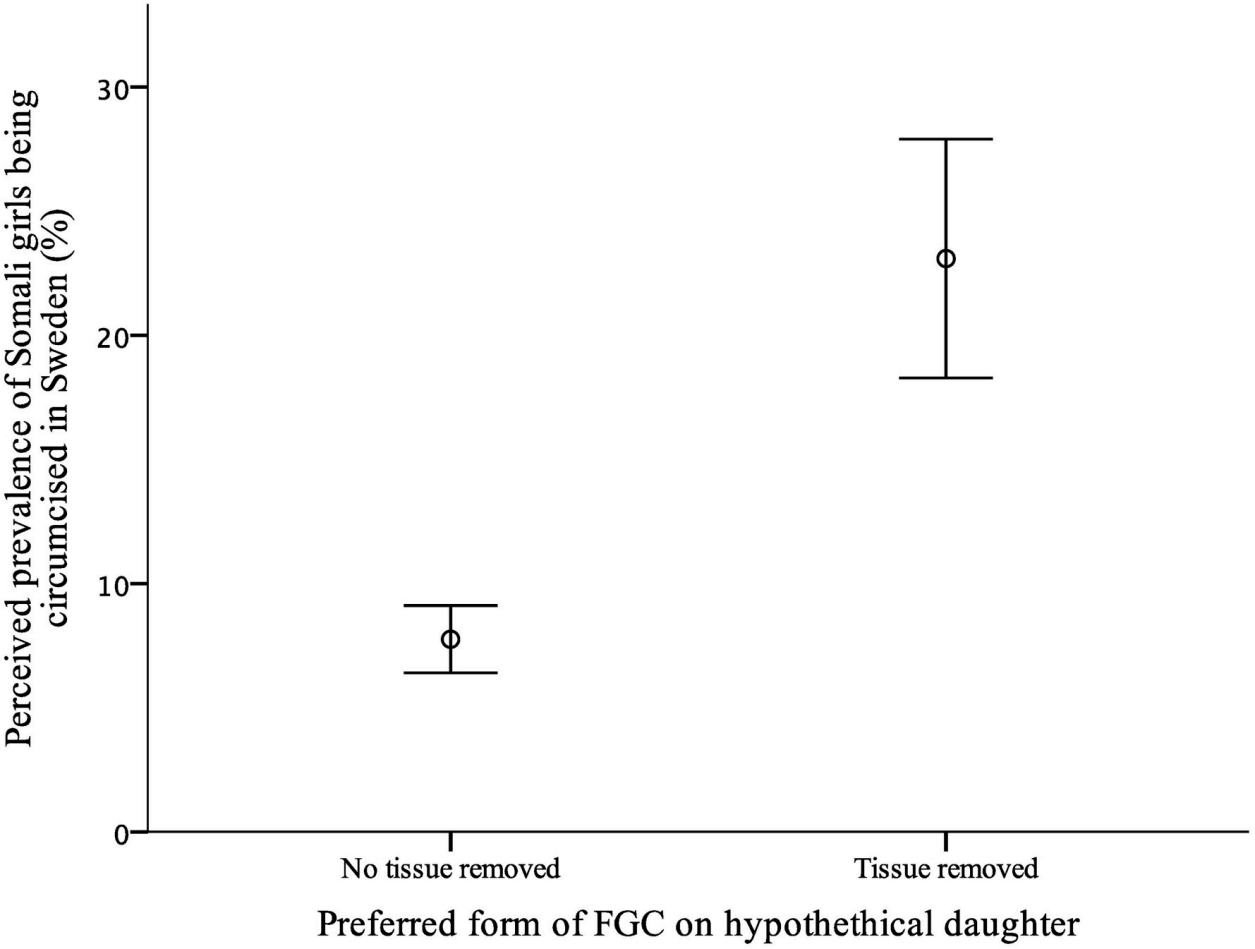
Participants preferred form of FGC on their own (hypothetical) daughter and their assessment of how many Somali girls get circumcised in Sweden (mean with 95% CI). Welch’s *t*-test: *p* < 0.001.

Swedish Somali men and women reported that they wanted the following forms of FGC to be performed on their (hypothetical) daughter: remain untouched 449/645 (70%), be pricked 94/645 (15%), have tissue removed without stitching 89/645 (14%), have tissue removed with stitching 3/645 (<1%), and have tissue removed with narrowing of the vaginal orifice 10/645 (2%). In Fig 2, the reported preferred form of FGC on a hypothetical daughter (dichotomised into ‘no tissue removed’ and ‘tissue removed’) is compared with how common the participant thought it was for Somali girls to be circumcised in Sweden (regardless of form of FGC). The group of Swedish Somalis who said they wanted tissue to be removed on a (hypothetical) daughter assessed that, on average, 23% (95% CI: 18.3–27.9) of the Swedish Somali community circumcise their daughters. This assessment was significantly higher (*p* < 0.001) compared with the group of Swedish Somalis opposing the removal of tissue, who assessed that, on average, 8% (95% CI: 6.4–9.1) of the Swedish Somali community circumcises their daughters.

### Hypothesis 3. The convention to circumcise in order to enhance marriageability is not found in a Swedish migration context

Marriage preference among Swedish Somali men in relation to women’s FGC status, and women’s presumptions about men’s preferences are presented in Table 4. The majority of Swedish Somali men, 92%, reported that they would prefer to marry someone without FGC or with pricking, which was in agreement with the presumption by most Swedish Somali women (90%). Only 6% of the men stated that FGC status did not matter, and a corresponding 5% of the women thought that FGC status was unimportant to men (Table 4).

**Table 4:** Marriage preference among Swedish Somali men in regard to women’s FGC status, and women’s presumptions of men’s preference.

	*n*/N	%
**Men prefer to marry**		
No tissue removed	301/327	92.0
Not circumcised	(249/327)	(76.1)
Pricked	(52/327)	(15.9)
Tissue removed	6/327	1.8
Does not matter	20/327	6.1
**Women think men prefer to marry**		
No tissue removed	277/309	89.6
Not circumcised	(210/309)	(68.0)
Pricked	(67/309)	(21.7)
Tissue removed	16/309	5.2
Does not matter	16/309	5.2

## Discussion

Our findings support the hypotheses that Swedish Somalis’ own propensity to support FGC is correlated with perceptions about what other Swedish Somalis prefer. Further, we found that marriage to a woman with no FGC was preferred over one with FGC, a finding also reported in qualitative studies with Somalis in Sweden and Norway [4,30].

There can be several explanations for our observed findings. It could be that programs aimed at abandonment of FGC–both in Somalia and in Sweden–as well as FGC not being the norm and legislated against in Sweden may have contributed to a shift in attitudes and reduced or removed the social pressure to perform FGC. Such attitude change after migration regarding FGC have been reported also in other European countries [31], and changes in the social norm towards FGC among migrants as a result of living in settings where non-circumcision is the norm has been reported [32,33]. It could also be that the observed attitudes towards FGC is a result of those migrating from Somalia being much less in favour of FGC than those who stayed in Somalia.

In Somaliland, 74% of men and women think that people in their community expect them to circumcise their daughters [34]. Living in Sweden could therefore also provide opportunities to create new social networks with women who are not cut, thereby shifting the reference group and reducing the social pressure to conform with the practice. Creating new social networks can be of importance as it has been found that kinship and collective thought in Somali culture strongly impact the lives of Somali individuals [35].

It is also a possibility that the participants were cautious when reporting their views towards FGC, and provided what they thought was the desired response, and the agreement we found between own attitudes towards FGC and perceived attitudes among others could be a reflection of the fact that individuals are more prone to report the same attitudes for themselves as for others as a way to rationalise their own views. However, we also found a tendency for individuals to report that other Swedish Somalis approved of FGC, while they themselves did not. This dissociation from the contemporary others has been interpreted by Jirovsky [36] as a way to separate oneself from individuals who are perceived as old-fashioned and uneducated as compared to being modern, and to emphasize that one has internalised the moral teachings of the campaigns against FGC. The difficulty of measuring how the group socially influences the individuals’ behaviour has been discussed in depth by Manski [37]. These challenges have also been raised by Mackie [38]. Thus, our findings should be interpreted with caution.

### Methodological considerations

A main concern in this study, as discussed above, is social desirability. There is a risk that the answers obtained to some extent reflect a social desirability bias; that is, that the participants answered what they thought the data collectors wanted to hear. However, we tried to minimise this risk by not asking directly whether they had a daughter, but instead asked them about a *hypothetical* daughter. Further, the data collectors were themselves Swedish Somali men and women, knowledgeable of the context, respected within the community, and not associated with any authority, thus, enabling a trusting relationship with the participants.

We collected data from men and women of different ages and living in different cities, providing a comprehensive understanding of FGC within the Swedish Somali context. However, as participants were recruited through purposive and, to some extent, snowball sampling, this may have caused a selection bias as well as resulted in a more uniform sample and thereby more uniform results. Although the questionnaire had been pilot-tested and translated and back-translated from English to Somali, translation from one language to another is a challenge and a possible limitation as some words may not have a corresponding one in the other language. As this was a cross-sectional study, causal inference cannot be established. Thus, we cannot determine whether the perceived behaviour within the Somali community affects the individuals’ propensity to support FGC or if it is the opposite. It may also be other factors that simultaneously correlates with the investigated variables, thereby influencing the correlation seen. It is also possible that some participants rationalised their own attitude by referring to the community as having the same attitude.

How to express attitudes on a VAS may have been understood differently between the participants, therefore, data collectors who had been trained in how to report attitudes on a VAS collected data through face-to-face interviews. Further, a schematic diagram describing roughly the different forms of FGC based on anatomy was provided. Our categorisation of pricking on the VAS was rather strict (1–10 mm); as a consequence, the number of participants supporting the removal of tissue (11–100 mm) may be an overestimation.

We chose to present data primarily divided into support of no removal of tissue compared to support of removal of tissue. We chose this anatomical classification as we found that some participants did not regard pricking as a form of FGC because in their view, for a practice to be considered as FGC, tissue needed to be removed. However, because the WHO classifies pricking as a form of FGC, we also presented data where attitudes supporting pricking are not grouped together with attitudes supporting no form of FGC.

## Conclusion

In this study, we have demonstrated how the group opinion, as perceived by the individual, may affect his or her own attitudes towards FGC. Thus, portraying FGC as being widely practised within migrant groups, in for example media reports, may have a negative impact on immigrants who are undecided whether to circumcise their daughters, as they may–based on their perception that ‘everybody else’ in their community practises FGC–‘tip over’ and opt for circumcision of their daughters [39]. For future research in this area, we recommend that surveys with questions about attitudes towards FGC among these groups in the wider host society are contrasted to surveys in the country of origin, to better pinpoint processes of attitude change in a migration context.

## Supporting information

S1 File Questionnaire in English. (DOCX)

S2 File Questionnaire in Somali. (DOCX)
