# Correction: Shifting perceptions of female genital cutting in a Swedish migration context

**DOI:** 10.1371/journal.pone.0229815

**Published:** 2020-02-26

**Authors:** Anna Wahlberg, Sara Johnsdotter, Katarina Ekholm Selling, Birgitta Essén

S3 File is omitted from the list of Supporting Information. It can be viewed below.

## Supporting information

S3 File Data set. (SAV)
