# Supplementary material for: Shifting perceptions of female genital cutting in a Swedish migration context
Source: PLoS One. 2019 Dec 4;14(12):e0225629. doi: 10.1371/journal.pone.0225629 (PMC6892496; doi:10.1371/journal.pone.0225629)
Supplement: S2 File — (DOCX) [file pone.0225629.s002.docx]

Somali SciLive

**Baseline questionnaire**

*Su'aal kasta mid kaliya ka dooro haddii aan si kale loo dhigin*

| AQOONSASHO | |
| --- | --- |
| 1. | Astaanta siredka ururka Soomaaliyeed ____________________ |
| 2. | Astaanta sirta ah ee ka qaybqaataha ____________________ |
| 3. | Magaca wareysi qaadaha ____________________ |
| 4. | Taarikhda/ bisha/ maalinta: ________/____/____ |
| 5. | Daqiiqadaha uu wareysiga socdey ____________________ |

| ASALKA QOFKA LA WAREYSANAYO | | | | | |
| --- | --- | --- | --- | --- | --- |
| 6. | Jinsi  Nin  Naag | | | | |
| 7. | **Sannadkee dhalatey?**  *Sida ku qoran basaboorkaada*  *Sannadka (SSSS):* ____________________ | | | | |
| 8. | **Waa imisa waqtiga aad Iswiden joogtay?**  2 sano ka yar  3 ilaa 4 sano  5 ilaa 9 sano  10 ilaa 14 sano  15 sano ka badan | | | | |
| 9. | **Waa maxay heerka wax barsho aad gaartay?**  Dugsi waxbarasho ma tagin/ ma aan dhameysan waxbarashada  Kugsi quraan kaliya Ayaan dhigtay  Dugsiga hoose dhexe (1 ilaa 9 sano)  Dugsiga sare (10 ilaa 12 sano)  Machad/ jaamacad | | | | |
| 10. | **Xagee ku barbaartay inta aadan iman Sweden?**  Magaalo weyn  Magaalo yar/ baadiyaha  Nolol reer guraa ah | | | | |
| 11. | **Waa maxay xaaladdaada guur?**  Kali  Xaas/ qof xiriir la leh (aan isguursan)  Garoob/ carmal laga dhintey | | | | |
| 12. | **Yaa gurigaada kugula nool?**  *Dhowr wax ayaad doorankartaa*  Kaligey  Ninkeyga/ marwadeyda/ lamaanaheyga  Carruur  Hooyo, aabe, sodogeyga, soddohdeyda  Qaraabo kale  Wax kale, qeex: ________________________________________________________________ | | | | |
| 13. | **Ma ka heshaa masaruuf ceyrta (försörjningsstöd) degmadaada?**  Haa  Maya | | | | |
| 14. | **Maxey tahay xaaladaad shaqo?**  Waqti buuxa Ayaan shaqeeyaa/ waqtiga bar Ayaan shaqeeyaa  Ka tirsan barnaamij hordhac, wax barasho, shaqo baradnimo, iwmoo ay dyarisay xafiiska shaqada (Arbetsförmedlingen)  Barashada luqadda swediska (SFI, Bilowga swediska, iwm)  Ardey (Qofka baranaya wax kale oo aan aheyn luqadda swedishka)  Howlgab/ Ku jira fasax xanuun dhaqtar u qoray/ fasax waalidka caruurta dhaqaalana lgu siiyo (föräldrapennig)  Aan shaqeynin | | | | |
| 15. | Waa maxay diintaadu?  Muslim  Masiixi/ krishtaan  Waxba aan aaminsaneyn  Wax kale | | | | |
| 16. | **Qiimee fahamkaada iyo isticmaalkaada luqadda Iswidhishka sida xaaladaha soo socda** | | | | |
|  |  | Waa liita | Waa Dhexdhexad | Si wanaagsan | Aad u Wanaagsan |
|  | (a) Fahamka aqbaarta ka baxda radiyahay iyo telefishinka |  |  |  |  |
|  | (b) In aad shirarka Iswedhishka ku hadasho |  |  |  |  |
|  | (c) In aad telefoonka kula xiriirto xafiisyada dowladda, (*sida,* in aad wacdo Qeybta Caafimaadka, Xafiiska amaanka bulshada, ama Xaruunta xiriirka shaqaalaha) |  |  |  |  |
|  | (d) In aad Iswedhishka buugaag ku aqrisato |  |  |  |  |
|  | (e) In aad diyaariso codsi shaqo qoraal ah |  |  |  |  |

| ASAL AHAAN, GUDNIINKA DHEDIGGA  Inta aanan ku weydiin suaasha hoose, tus sawirka (sawirka 1) ka jawaabaha noocyada kala duwan ee gudniinka dhidiga ah. U sharax noocyada kala duwan, iyo sida qofwalbaa uusan u raacsaneyn noocyada kala duwan ee gudniinka haweenka. Tus sawrika kale (sawirka 2) unna sharax kani xitaa in uu looyahay xulasho aan waxba la sameyneyn.  Ku heyso sawiradaa ka qeybgalaha hortiisa inta aad wareysaneyso oo idil, iyaga u gudbi marka ay suaasha ka bilaabaneyso sawirka 1 amma sawirka 2.  Suaalaha xariijinta baaxadda leh, jawaabuhu waa in uu laftiisa xariijin ku qoro warqadda sulaaha.  Hubso in aad u sharaxday jawaabaha arigtidiisa iyo ra yigiisa amma rayigeeda in lagu kalsoon yahay, markaasina in aynaan jirin wax sax ah amma khalad ah. | |
| --- | --- |
| 17. | Eeg sawirka 1: Keebaad ku xiriirn laheyd gudniinka gabdhaha?  *Notera: Med denna fråga menas vilka ingrepp de tycker hör till begreppet “kvinnlig omskärelse”.*  *Qor: jahee tilmaanta*  Dhiijin, aan Cad la jaro  cad la goyn lana xiro |
| Sharax, marka aan isticmaaleyno ereyga gudniinka dhidiga ee suaasha hoose waxaa ku jira noocwalba. | |
| 18. | **Qof ka mid ah qoyskaaga ma maray gudniinka dhidiga ah?**  Haa  Maya  Magaranayo |
| 19. | Weydii haweenka kaliya: Adiga laftaada ma lagu guday, hadii lagu guday, maxa lagugu sameeyay markaa?  *Qor: Eeg sawirka 2.*  Haa: Dhiijin aan cad la jarin  Haa: Cad ayaa la jaray  Haa: Cad ayaa la jaray, qodobaa la tolay  Haa: Cad ayaa la jaray waana leysku xiray  Maya  Magaranayo  N/A |
| 20. | Somalia dhexdeeda, waligaa ma heshay warbixin dhanka bulshada oo ku aadan gudniinka dhediga amma gabdhaha sida (video, Tv, wargeysyo, internetka)?  *Qor: Eeg sawirka 2.*  Haa  Hadii ay haa tahay, ma aheyd warbixinta guud ahaan:  Taageereyso:  Dhiijin aan cad la jarin  Cad ayaa la jaray  Cad ayaa la jaray qodobaa la tolay  Cad ayaa la jaray waana leysku xiray  Aan taageereynin:  Dhiijin aan cad la jarin  Cad ayaa la jaray  Cad ayaa la jaray qodobaa la tolay  Cad ayaa la jaray waana leysku xiray  Maya |
| 21. | Swiden dhexdeeda, waligaa ma heshay warbixin dhanka bulshada oo ku aadan gudniinka dhediga amma gabdhaha sida (video, Tv, wargeysyo, internetka)?  *Qor: Eeg sawirka 2.*  Haa  Hadii ay haa tahay, ma aheyd warbixintu guud ahaan:  Taageereyso:  Dhiijin aan cad la jarin  Cad ayaa la jaray  Cad ayaa la jaray qodobaa la tolay  Cad ayaa la jaray waana leysku xiray    Aan taageereynin:  Dhiijin aan cad la jarin  Cad ayaa la jaray  Cad ayaa la jaray qodobaa la tolay  Cad ayaa la jaray waana leysku xiray  Maya |
| 22. | **Adiga laftaada weliga maka qeybqaadatey koox, urur, ama olole ka soo horjeeda guddniinka haweenka?**  *Qor: ka qeybqaadashada daraasaddan Somali SciLive intervention maha in loo diiwan galiyo (haa Sweden)*  Haa, Somaliya  Haa, Iswidhen  Haa, labadaba Somaliya iyo Iswidhen  Maya |
| GUDDNIINKA DHEDDIGA, GABADHAADA | |
| 23. | Eeg sawirka 2: ma garaneyno hadii aad gabar amma innan leedahay. Laakiin aan iska niraahdo waxaad leedahay gabar. Maxaad sameyn lahayd?  Waxba ma Cad ayaa lajaray  sameyn waana leysku  laheyn xiray |
| 24. | Somalida ku nool Sweden imiisa ayey kula tahay in ay gabdhahooda gudi lahaayeen  Qofna Qof walba |
|  | |
| GUDDNIINKA DHEDDIGA, SIDA LOO ARKO | |
| 25. | Eeg sawirka 2: Maxay kula tahay in la ogolaankaro in la sameeyo?  *Notera: Här ska den intervjuade svara på vilka typer av ingrepp han/hon tycker är acceptabla att utföra.*  *Qor: Jahee tilmaanta*  Waxba Cad la jaro,  oo leysku xiro |
| 26. | Eeg sawirka 2: Maxay kula tahay ragga somaliyed ee Swiden ku nool intooda badan in ay ku fekerayaan in la ogolankaro in la sameeyo?  Qor: Jahee tilmaanta  Waxba Cad la jaro,  oo leysku xiro |
| 27. | Eeg sawirka 2: Maxay kula tahay dumarka somaaliyed ee ku nool sweden badidooda in la ogolaankaro in la sameeyo?  Qor: Jahee tilmaanta  Waxba Cad la jaro,  oo leysku xiro |
| 28. | Eeg sawirka 2: Maxay kula tahay adigoo ku saleynaayo diintaada in la ogol yahay in la sameeyo?  *Qor: Jahee tilmaanta*  Waxba Cad la jaro,   oo leysku xiro |
| 29. | Eeg sawirka 1: Gudniinka goorta amma hadii uu abadkiiba u geystaa gabdhaha yar iyo haweenka dhibaato caafimaad daro fog?  Qor: Jahee tilmaanta  Dhiijin, aan Cad la jaro,   cad la jari oo leysku xiro  Marna |
| 30. | Eeg sawirka 1: Goorma, amma hadii abadkiiba uu noqdaa gudniinku xadgudubka xaquuqda caruurta?  *Qor: Jahee tilmaanta*  Dhiijin, aan Cad la jaro,   cad la jari oo leysku xiro    Marna |
| 31. | Eeg sawirka 2: In laguu arko in aad tahay qof sharaf leh adigoo gabar dhalinyaro ah oo aan weli guursan, maxaa loo baahanyahay in la sameeyo aragti ahaantaada?  *Qor: jahee tilmaanta*  Waxba Cad la jaro,  oo leysku xiro |
| 32. | Waa maxeey faa iidooyinka ay gabdhuhu ka heli karaan Gudniinka? *Dhowr wax ayaad doorankartaa*  Nadaafadda/Nadaafadda caafimaad  Mujtamaca oo oggol  Rajo guur fiican  Ilaalin bikranimada/ Joojineyso galmada  Raaxada galmada oo lagu yareeyo  Diinta oo raali ka ah  Wax kale, qeex:____________________________________________________________________  Wax faaido ah ma laha |
| 33. | **Waa maxeey faa iidooyinka gabdhuhu ku heli hadii aysan gudneeyn?** *Dhowr wax ayaad doorankartaa*  Dhibaatooyinka caafimadka oo ku yarada  Kafogaanshada xanuunka  Raaxada galmada oo u macaato iyada  Raxaada galmada oo u macaato asiga  Raaco Diinta  Wax kale, qeex: ____________________________________________________________________  Wax faaido ah ma laha |
| 34. | Waxaa jira dad doonaya gudniinka haweenka in la cirib tiro, waxaa kaloo jira dad doonaya in gudniinka gabdhaha sii socdo, qeybaha soo socda maxaad ka dooneysaa in ay sii socdaan?  *Qor: Fursado dhowr ah ayaa la xulan karaa. Eeg sawirka 1.*  Dhiijin aan cad la jarin  Cad ayaa la jaray  Cad ayaa la jaray qodobaa la tolay  Cad ayaa la jaray waana leysku xiray  Gedigoodba waa in la cirib tiro |
| 35. | **Ma kula tahay in ay fiicantahay in ragga ka qeybqaataan doodda ku saabsan gudniinka dheddiga?**  Haa  Maya |
| 36. | Weydii ragga uun: Guursigaada, ma doorbideysaa haween gudan misse waxaad doorbideysaa mid aan gudneyn?  *Qor: Eeg sawirka 2.*  Aan gudneyn  Gudan: dhiijin aan cad la jarin  Gudan: cad la jaray  Gudan: cad la jaray qodobaa la tolay  Gudan: cad la jaray oo leysku xiray  Arintaa oo dhan maqabo  N/A |
| 37. | Weydii haweenka oo kaliya: ma kula tahay ragga somaliyeed in ay door bidayaan in ay guursadaan haween gudan missw mid aanan gudneyn?  *Qor: Eeg sawirka 2.*  Aan gudneyn  Gudan: dhiijin aan cad la jarin  Gudan: cad la jaray  Gudan: cad la jaray qodobaa la tolay  Gudan: cad la jaray oo leysku xiray  Arintaa oo dhan maba qabaan  N/A |
| GUDDNIINKA DHEDDIGGA, AQOON | |
| 38. | Eeg sawirka 2: Maxaa sharci ka ah Sweden oo la sameynkaraa?  *Qor: Jahee tilmaanta*  Waxba Cad la jaray,   oo leysku xiro  Magaranayo |
| 39. | Ma sharci baa ku noolaashaha Sweden in gabar loo kaxeeyo Dibada si’ loo gudo?  Haa  Maya  Magaranaayo |

| GUDDNIINKA LABKA, ASALKA | |
| --- | --- |
| 40. | Weydii ragga oo kaliya: Adiga lafa ahaantaada ma lagu guday?  Haa  Maya  Magaranayo  N/A |
| 41. | Ma u aragtaa in ay jirto sabab loo suaalo gudniinka wiilasha?  *Notera: Här ska den intervjuade svara på om han/hon ser några skäl till att inte utföra manlig omskärelse, om han/hon ser något skäl till att ifrågasätta utförandet av manlig omskärelse.*  Haa  Maya |
| 42. | **Dadka qaarkood ayaa sheegaya in guddniinka wiilasha uu yahay ku xadgudub xuquuqda carruurta, ma ku raacsantahay?**  Haa  Maya |
| 43. | Marka loo eego caafimaadka jirka, maxaad ku tilmaami laheyd waxyeelada gudniinka labka?  *Notera: Om sträcket dras precis i mitten innebär det att den intervjuade varken ser några fördelaktiga eller skadliga effekter av manlig omskärelse. Om strecket dras mer till vänster innebär det att den intervjuade tycker att det finns fler fördelaktiga effekter, och om strecket dras mer till höger så tycker den intervjuade att det finns fler skadliga effekter.*  Faaido kaliya Waxyeelo kaliya |
| 44. | **Ra`yi ahaantaada dhaqanka guddniinka gabdhaha iyo tan wiilasha ma isu dhigmaan?**  *Qor: Fursadu waa dhowr wax oo la dooran karo. Eeg sawirka 2.*  Haa, dhiijinta oo aan cad la jarin waxay u dhigantaa sida tan wiilasha  Haa, marka cad la jaro waxay u dhigantaa sida tan wiilasha  Haa, marka cad la jaro oo la tolo waxay u dhigantaa sida tan wiilasha  Haa, marka cad la jaro oo leysku xiro amma la daboolo waxay u dhigantaa sida tan wiilasha  Maya, maya midna uma dhigmo sida tan wiilasha |

| RAASUMAALKA MUJTAMACA  Ka hor waydiinaya su'aalo hoose, sharxa jawabta in aad wediin doonto su'aalo ku saabsan isaga / iyada nolol maalmeedka ee Sweden. | | | | | |
| --- | --- | --- | --- | --- | --- |
| **45.** | **Go,aan ka gaar bayaannadaan soo socda** | | | | |
|  |  | Kuma raacsani haba yaraatee | Kuma raacsani | Waan ku raacsanahay | Waan ku raacsanahay si' buuxda |
|  | (a) Dadka intooda badan wey kaa faaideysanayan haddii ay fursad u helaan |  |  |  |  |
|  | (b) Dadka intooda badan waxey isku dayaan in ay caadil ahaadaan |  |  |  |  |
|  | (c) Waxaad aamini karta dadka intooda badan |  |  |  |  |
|  | (d) Waa in aad loo taxadaro marka dad kale lala dhaqmayo |  |  |  |  |
| **46.** | **Muddada 12 bilood ah ee ugu dambeeyey ma...**  *Qor: Waxyaabaha hoos ku qoran waxaa lagu dabiqi kara oo kaliya waqtiga la joogey Iswidhen. Jawaabo dhowr ah ayaa la dooran kara.*  Ka qeybqaadatey waxbarasho (studiecirkel)  Ka qeybqaadatey shir urur shaqaale  Ka qeybqaadatey shir urur Soomaaliyeed  Ka qeybqaadatey shir ururo kale  Tagtey masraxiyad/shaneemo  Tagtey munaasabad/dhaqdhaqaaq dhaqameed  Ka qeybqaadatey munaasabad diineed  Tagtey tartan-ciyaareed  Ka qeybqaadatey dabaaldeg maalinta xornimada Somaliya  Ka qeybqaadatey banaanbax nooc kastaba ha ahaadee  Tagtey munaasabad fagaaro laysku yimaado, tusaale riwaayad ama musig, maaweelo i.w.m.  Ka qeybqaadatey kulan weyn ee qaraabadaada  Tagtey xaflad gaar ama xaflad aroos/nikaax  Midna waxyaabaha kor ku qoran oo dhan | | | | |
| **47.** | **Howlaha bulshada waqtiyada aad ka qeybqaadatay dadka aad kala qeybqaadatay howshaa ma waxay ahaayeen dad aad isku asal tihiin (sida Jinsiyadda, rag amma dumar.tacliin- amma isku wadan)?**  *Qor: Dooro jawaabaha*  Dadka ka qeybqaatey oo dhan waxey ahaayen dad aan isku asal nahay  Dadka ka qeybqaatey badankood waxey ahaayen dad aan isku asal nahay  Dadka ka qeybqaatey qiyaas kala bar waxey ahaayen dad aan isku asal nahay  Dadka ka qeybqaatey badidooda ahaayen dad aan isku asal ahayn  Dadka ka qeybqaatey oo dhan waxey ahaayen dad asalkoodu kala duwanyahay | | | | |
| **48.** | **Dadka asalkooda (sida jinsi- tacliin, ama waddanka uu u dhashey) saameyn maku yeelaneysaa xadka aad dadkaas amini karto mathalan, kartida ay u leeyhihiin waxayaabaha ay ka hadlayaan?**  *Qor: Hal jabaab ka dooro*  Waxaa ku kalsoonahay dadka aan isku asal nahay  Waxaa ku kalsoonahay dadka aan isku asal nahay si’ ka badan dadka kale  Waxaa ku kalsoonahay dadka aan isku asal nahay xooga ka badan dadka kale  Waxaa ku kalsoonahay dadka aan isku asal nahay si la mid ah dadka kale  Waxaa ku kalsoonahay dadka aan isku asal nahay si ka ka yar dadka kale | | | | |
| **49.** | **Sidee igu wanaagsan ayey isu waafaqsanyihiin fikradahaada iyo waxyaabaha aad qiimeyneyso iyo kuwa dadka kale ee aad isku asalka tihiin (sida jinsi- labood ama dheddig-, tacliin, ama waddanka uu u dhashey)?**  *Qor: Hal jabaab ka dooro*  Fikradaheyga iyo waxyaabaha aan qiimeynayo iyo kuwa dadka aan isku asalka nahay waa isku mid  Fikradaheyga iyo waxyaabaha aan qiimeynayo badankooda iyo kuwa dadka aan isku asalka nahay waa isku mid  Fikradaheyga iyo waxyaabaha aan qiimeynayo iyo kuwa dadka aan isku asalka nahay qiyaasti kala bar waa isku mid  Fikradaheyga iyo waxyaabaha aan qiimeynayo waa ay kala duwanyihiin marka la barbar dhigo kuwa dadka aan isku asalka nahay | | | | |
